# Climate change heterogeneity: A new quantitative approach

**DOI:** 10.1371/journal.pone.0317208

**Published:** 2025-01-28

**Authors:** María Dolores Gadea Rivas, Jesús Gonzalo

**Affiliations:** 1 Department of Applied Economics, University of Zaragoza, Zaragoza, Spain; 2 Department of Economics, University Carlos III, Getafe, Spain; Universitat de Barcelona, SPAIN

## Abstract

Climate change is a spatial and temporarily non-uniform phenomenon that requires understanding its evolution to better evaluate its potential societal and economic impact. The value added of this paper lies in introducing a quantitative methodology grounded in the trend analysis of temperature distribution quantiles to analyze climate change heterogeneity (CCH). By converting these quantiles into time series objects, the methodology empowers the definition and measurement of various relevant concepts in climate change analysis (warming, warming typology, warming amplification and warming acceleration) in a straightforward and robust testable linear regression format. It also facilitates the introduction of new testable concepts like warming dominance to compare (globally or partially) the warming process experienced by different regions. Furthermore, the methodology holds the added significance of concurrently encompassing both temporal and spatial dimensions in temperature analysis, owing to the close alignment between unconditional quantiles and latitude measures. Applying our quantitative methodology for the period 1950-2019 to the Globe (2192 stations) and Spain (30 stations) as a benchmark region, we find that both experience a distributional warming process (beyond the standard average) but of very different types. While the Globe experiences a stronger warming in the lower temperatures than in the upper ones, Spain evolves from equal warming in the whole distribution toward a stronger warming in the upper quantiles (similar to the warming process experienced in the African continent). In the two cases, the warming process accelerates (non-linear behavior) over time and is asymmetrically amplified. Overall, although both the Globe and Spain suffer an equivalent warming process in the median (mean) temperature, Spain’s warming dominates the Globe in the upper quantiles and is dominated in the lower tail of the global temperature distribution that corresponds to the Arctic region. Our climate change heterogeneity results open the door to the need for a non-uniform causal-effect climate analysis that goes beyond the standard causality in mean and for a more efficient design of the mitigation-adaptation policies. In particular, the heterogeneity found suggests these policies should contain a common global component and a clear local-regional idiosyncratic element. The latter is usually more straightforward to implement.

## 1 Introduction

All the assessment reports (AR) published by the Intergovernmental Panel of Climate Change (IPCC) show that there is overwhelming scientific evidence of the existence of global warming (GW). It is also well known that climate change (CC) is a non-uniform phenomenon. What is not so clear is the degree of heterogeneity across all the regions in our planet. In fact, an important part of the Sixth Assessment Report (AR6) published by the IPCC in 2021–2022 [1–3] is dedicated to this issue: climate (warming) heterogeneity. Chapter 10 in [1] links global to local regional climate change ending with an Atlas application where different regional climates can be graphically compared. [2] dedicates several chapters to describe climate change in different continents. Chapter 13 in [3] focuses on different national and sub-national mitigation-adaptation policies. Our paper introduces a new quantitative methodology that builds on that described in [4] (GG2020, henceforth) to characterize, measure and test the existence of such climate change heterogeneity (CCH). This is done in three steps. First, we introduce a warming typology (*W1*, *W2* and *W3*) based on the trending behavior of the quantiles of the temperature distribution of a given geographical location. Second, we define in a testable format the concepts of warming acceleration and warming amplification. These concepts help to characterize (more ordinally than cardinally) the warming process of different regions. And third, we propose the new concept of warming dominance (WD) to establish when region *A* suffers a stronger warming process than region *B*. WD takes into account the whole distribution and not only the average temperature. The mean temperatures of both regions commonly follow similar trends; however, specific quantiles reveal distinct warming patterns.

We have chosen Spain as a benchmark geographical location because, as the AR6 report states “… Spain is fully included in the Mediterranean (MED) Reference Region, but is one of the most climatically diverse countries in the world…”. This fact opens up the possibility of studying warming heterogeneity (WH) from Spain to the Globe (outer heterogeneity, OWH) and also from Spain to some of its regions represented by Madrid and Barcelona (inner heterogeneity, IWH).

The quantitative methodology proposed in this paper relies on GG2020, where the different distributional characteristics (moments, quantiles, inter quantile range, etc.) of the temperature distribution of a given geographical location are converted into time series objects. By doing this, we can easily implement and test all the concepts involved in the three steps with historical observational data. Our quantitative methodology is well suited to deal with the non-stationarities present in climate data. Also by focusing on the trend behavior of the different characteristics, the proposed tests via regression models are robust to many types of possible misspecifications (heteroskedasticity, serial correlation, etc) present in the error regression terms.

It complements the climate literature of the past decade where several papers analyze climate change beyond the average temperature by considering the evolution of the whole temperature distribution. [5] tests a shifting in the whole temperature probability density, and [6] uses quantile trend regression for the average temperature process to detect different quantile slopes. The former needs to use a long period of time (30 years) to estimate the densities and this may face some problems when the temperature data is non-stationary during that period. The latter estimates the density evolution of the mean temperature process and this differs from the evolution of the whole unconditional temperature distribution analyzed with our methodology. [7] takes the perspective of climate as a constantly changing distribution, evaluates the relative changes between different quantiles of such distributions and between different geographical locations for the same quantiles. This is done graphically but with no statistical testing of the trend trajectory results. [8] uses a multidimensional ensemble empirical mode decomposition (MEEMD) for time series analysis such that the temperature at a grid point is decomposed into oscillatory components and a residual trend (the object of interest). One this trend is extracted it can be detected which region (latitude) is warming more in mean and if there has been some acceleration. With MEEMD is not so clear how to statistically compare the whole temperature distribution of different regions or how to determine the type of warming inside a given region.

Summarizing, the value added of this paper lies in introducing a quantitative methodology grounded in the trend analysis of temperature distribution quantiles to analyze climate change heterogeneity (CCH). By converting these quantiles into time series objects, the methodology empowers the definition and measurement of various relevant concepts in climate change analysis (warming, warming typology, warming amplification and warming acceleration) in a straightforward and robust testable linear regression format. It also facilitates the introduction of new testable concepts like warming dominance to compare (globally or partially) the warming process experienced by different regions. Furthermore, the methodology holds the added significance of concurrently encompassing both temporal and spatial dimensions in temperature analysis, owing to the close alignment between unconditional quantiles and latitude measures. More complex quantitative methods in spatial statistics and functional analysis exist that can capture both dimensions; however, our approach provides greater simplicity and robustness.

An outline of the results is as follows. Spain and the Globe present a clear warming process; but it evolves differently. Spain goes from a warming process where lower and upper temperatures share the same trend behavior (the interquantile range, *iqr*, is maintained constant over time, warming type *W1*) to one characterized by a larger increase in the upper temperatures (*iqr* increases over time, warming type *W3*). In contrast, the Globe as a whole maintains a stable warming type process characterized by lower temperatures that increase more than the upper ones (*iqr* decreases in time). Similar results for Central England are found in [4] and for US in [9]. In our typology, this constitutes a case of warming type *W2*. Climate heterogeneity can go further. For instance, within Spain we find that Madrid is of type *W3* while the warming process of Barcelona is of type *W1*. This is in concordance with the Madrid climate being considered a Continental Mediterranean climate while Barcelona is more a pure Mediterranean one.

The proposed warming typology (*W0*, *W1*, *W2* and *W3*), although dynamic, is more ordinal than cardinal. In this paper, the strength of a warming process is captured in the second step by analyzing its acceleration and its amplification with respect to a central tendency measure of the temperature distribution. Acceleration and amplification contribute to the analysis of warming heterogeneity. Acceleration is one of the non-linear aspects of climate change (see [10]). The acceleration in the Globe is present in all the quantiles above *q30* while in Spain it already becomes significant above the 10^*th*^ quantile. We find an asymmetric behavior of warming amplification; in Spain (in comparison with the Globe mean temperature) this is present in the upper temperatures (above the 80^*th*^ and 90^*th*^ quantiles) while in the Globe the opposite occurs (below the 20^*th*^ and 30^*th*^ quantiles). Within Spain, Madrid and Barcelona also behave differently in terms of acceleration and amplification. Overall, warming in Spain dominates that of the Globe in the upper quantiles but it is dominated in the lower tail of the distribution corresponding to the Arctic region. Between Madrid and Barcelona there is also a partial WD. Madrid WD Barcelona in the upper part of the distribution and Barcelona WD Madrid in the lower one. Interestingly, in both cases, Spain-Globe, and Madrid-Barcelona there is no dominance in the median (mean). Our observational results have to be seen as complementary to the ones obtained with climate models simulations.

The existence of a clear heterogeneous warming process opens the door to the need of a new non-uniform causal (effect) research. One that goes beyond the standard causality in mean analysis (see [11]). In this way, we can allow for different climate sensitivity parameters (*CO*2 may affect more the lower temperature quantiles than the upper ones). It is also possible that the temperature effects on productivity, income inequality, health, etc., depend on other different distributional characteristics apart from the average. CCH also suggests that in order for the mitigation-adaptation policies to be as efficient as possible they should be designed following a type of common factor structure: a common global component plus an idiosyncratic local element. This goes in the line with the results found in [12–14]. Future climate agreements should clearly have this CCH into account. An important by-product of our warming heterogeneity results is the increase that this heterogeneity can generate in the public awareness of the GW process. A possible explanation for that can be found in the behavioral economics work by [15], in the results of the European Social Survey analyzed in [16] or in the psychology survey by [17].

The rest of the paper is organized as follows. Section 2 describes our basic climate quantitative methodology. Section 3 presents a brief description of the temperature data from the Globe and Spain. Section 4 addresses the application of our quantitative methodology in the cross-sectional version (temperatures measured monthly by stations in an annual interval) to the Globe and (versus) Spain. It also reports the results of applying the methodology using a purely temporal dimension (local daily temperature on an annual basis) for two representative stations in Spain (Madrid and Barcelona, empirical details in the S1 Appendix). Section 5 offers a comparison and interpretation of the results. Finally, Section 6 concludes the paper.

## 2 Climate quantitative methodology

In this section, we first briefly summarize the econometric quantitative methodology introduced in GG2020 that will be used to analyze the Global and Local warming processes. Second, we introduce the climate concepts that will be applied to describe quantitatively the different warming processes: “warming”, “warming typology”, “warming acceleration”, “warming amplification” and “warming dominance”. The value added of this quantitative methodology is that all the concepts are defined in a simple robust testable linear regression format.

### 2.1 GG2020 approach

Following GG2020, Warming is defined as an increasing trend in certain characteristics of the temperature distribution. More precisely:

**Definition 1**. *(Warming)*: *Warming is defined as the existence of an increasing trend in some of the distributional characteristics measuring the central tendency or position (quantiles) of the temperature distribution*.

An example is a deterministic trend with a polynomial function for certain values of the *β* parameters *C*_*t*_ = *β*_0_ + *β*_1_*t* + *β*_2_*t*^2^ + … + *β*_*k*_*t*^*k*^, as well as, an integrated process of order one (*I*(1)). An *I*(1) process is the accumulation of an *I*(0) process. Our definition of an I(0) process follows [18]. A stochastic process *Y*_*t*_ that satisfies *Y*_*t*_ − *E*(*Y*_*t*_) $=\sum_{i=1}^{\infty }\Psi_{i}\varepsilon_{t-i}$ is called I(0) if $\sum_{i=1}^{\infty }\Psi_{i}z^{i}$ converges for |*z*| < 1 + *δ*, for some *δ* > 0 and $\sum_{i=1}^{\infty }\Psi_{i}\neq 0$, where the condition *ε*_*t*_ ∼ iid(0,*σ*^2^) with *σ*^2^ > 0 is understood.

In GG2020 temperature is viewed as a functional stochastic process *X* = (*X*_*t*_(*ω*), *t* ∈ *T*), where *T* is an interval in R, defined in a probability space (Ω, ℑ, *P*). A convenient example of an infinite-dimensional discrete-time process consists of associating $\xi =(\xi_{n},n\in R_{+})$ with a sequence of random variables whose values are in an appropriate function space (see [19]). This may be obtained by setting

$$\begin{array}{c} X_{t}\left(n\right)=\xi_{tN+n},\;0\leq n\leq N,\;t=0,1,2,\ldots ,T \end{array}$$ (1)

so *X* = (*X*_*t*_, *t* = 0, 1, 2, …, *T*). If the sample paths of *ξ* are continuous, then we have a sequence *X*_0_, *X*_1_, …. of random variables in the space *C*[0, *N*]. The choice of the period or segment *t* will depend on the situation in hand. In our case, *t* will be the period of a year, and *N* represents cross-sectional units or higher-frequency time series.

We may be interested in modeling the whole sequence of **G** functions, for instance the sequence of state densities (*f*_1_(*ω*), *f*_2_(*ω*), …, *f*_*T*_(*ω*)) as in [20, 21] or only certain characteristics (*C*_*t*_(*w*)) of these **G** functions, for instance, the state mean, the state variance, the state quantile, etc. These characteristics can be considered time series objects and, therefore, all the econometric tools already developed in the time series literature can be applied to *C*_*t*_(*w*). This resembles the quantile curve estimation analyzed in [22, 23]. With this characteristic approach we go from Ω to $R^{T}$, as in a standard stochastic process, passing through a **G** functional space:

$$\underset{\left(w\right)}{\Omega }\overset{X}{\to }\underset{X_{t}\left(w\right)}{G}\overset{C}{\to }\underset{C_{t}\left(w\right)}{R}$$

Going back to the convenient example and abusing notation (*X*_*t*_(*n*) = *X*_*tn*_ and *n* a natural number), the stochastic structure can be summarized in the following array:



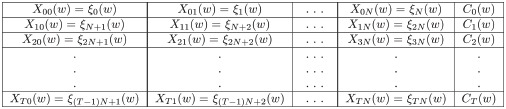

(2)


Throughout this paper, similarly to the assumptions made in [20, 23, 24], we assume that in each period, *t*, the stochastic functional process *X* = (*X*_*t*_(*ω*), *t* ∈ *T*) satisfies certain regularity conditions (local stationarity, strong mixing conditions, etc.) and there are sufficient temporal or cross-sectional observations (*N* → ∞) for these characteristics to be estimated consistently. The main characteristics analyzed in this paper are moments and quantiles (*q*_*τ*_, 0 < *τ* < 1) of the temperature distribution.

After the development of the proposed theoretical core, we are in a position to design tools to approach the empirical strategy. The following subsection describes each of them.

### 2.2 Empirical tools: Definitions and tests

One of the objectives of this section is to provide a simple test to detect the existence of a general unknown trend component in a given characteristic *C*_*t*_ of the temperature process *X*_*t*_. To do this, we need to convert Definition 1 into a more practical definition.

**Definition 2**. *(Practical trend definition)*: *A characteristic C*_*t*_ *of a functional stochastic process X*_*t*_ *contains a trend if in the LS regression*,

$$\begin{array}{c} C_{t}=\alpha +\beta t+u_{t},\;t=1,\ldots ,T, \end{array}$$ (3)

*β* = 0 *is rejected*.

GG2020 shows that a simple *t* − *test* (*H*_0_ : *β* = 0, *H*_1_ : *β* ≠ 0) that asymptotically follows a normal distribution under *H*_0_ is able to detect most of the existing deterministic trends (polynomial, logarithmic, exponential, etc) and also the trends generated by any of the three standard persistent processes considered in the literature (see [25]): (i) fractional or long-memory models (1/2 < *d* < 3/2); (ii) near-unit-root AR models; and (iii) local-level models.

Several remarks are relevant with respect to this definition: (i) regression (3) has to be understood as the linear LS approximation of an unknown trend function *h*(*t*) (see [26]); (ii) the parameter *β* is the *plim* of ${\hat{\beta }}_{ols}$; (iii) if the regression (3) is the true data-generating process, with *u*_*t*_ ∼ *I*(0), then the OLS $\hat{\beta }$ estimator is asymptotically equivalent to the GLS estimator (see [27]) and the *t*−*test*(*β* = 0) is *N(0,1)*; (iv) in practice, in order to test *β* = 0, it is recommended to use a robust HAC version of *t*_*β* = 0_ (see [28]); and (v) this test only detects the existence of a trend but not the type of trend. Notice also that in (3) we could be totally agnostic about *u*_*t*_ being *I(0)* or *I(1)*. In this case following [29] we can estimate the model by Feasible Generalized Least Squares and construct a similar *t-stat* of *β* = 0 that still will follow a *N(0,1)*. This method depends on a tuning parameter (how closes is *u*_*t*_ of being *I(1)*). To avoid that, in this paper, we follow an alternative approach. We pre-test the temperature data for unit roots, once they are rejected we proceed as if *u*_*t*_ in 3 is *I(0)*.

For all these reasons, in the empirical applications we implement Definition 2 by estimating regression (3) using OLS and constructing a HAC version of *t*_*β*=0_ [30]. In the definition of *C*_*t*_ we can consider any distributional characteristics as time series objects. This set includes not only the quantiles that constitute the distribution, but also other characteristics that may be of interest, enabling a much more comprehensive analysis of temperature than is achievable with the mean alone.

These linear trends can be common across characteristics indicating similar patters in the time evolution of these characteristics.

**Definition 3**. *(Co-trending)*: *A set of m distributional characteristics* (*C*_1*t*_,*C*_2*t*_,…,*C*_*mt*_) *do linearly co-trend if in the multivariate regression*

$$\begin{array}{c} \left(\begin{array}{c} C_{1t}\\ \ldots \\ C_{mt} \end{array})=(\begin{array}{c} \alpha_{1}\\ \ldots \\ \alpha_{m} \end{array})+(\begin{array}{c} \beta_{1}\\ \ldots \\ \beta_{m} \end{array})t+(\begin{array}{c} u_{1t}\\ \ldots \\ u_{mt} \end{array}\right) \end{array}$$ (4)

*all the slopes are equal*, *β*_1_ = *β*_2_ = … = *β*_*m*_.

This definition is slightly different from the one in [31].

This co-trending hypothesis can be tested by a standard Wald test (HAC version) with *H*_0_ : *β*_1_ = *β*_2_ = … = *β*_*m*_ and *H*1 : ∃*β*_*i*_ ≠ *β*_*j*_, *i* ≠ *j* ∈ 1, 2, …, *m*. This test follow asymptotically a $\chi_{m-1}^{2}$ distribution.

When *m* = 2 an alternative linear co-trending test can be obtained from the regression

$$\begin{array}{c} C_{it}-C_{jt}=\alpha +\beta t+u_{t} \end{array}$$

*i* ≠ *j* *i*, *j* = 1, …, *m* by testing the null hypothesis of *β* = 0 vs *β* ≠ 0 using a simple *t*_*β* = 0_ test.

Notice that this test does not suffer of spurious co-trending. In a situation where *C*_*it*_ and *C*_*jt*_ were two non-cointegrated *I*(1) processes, the test would reject the null hypothesis of *β* = 0 and conclude that both characteristics do not co-trend.

Climate classification is a tool used to recognize, clarify and simplify the existent climate heterogeneity in the Globe. It also helps us to better understand the Globe’s climate and therefore to design more efficient global warming mitigation policies. The prevalent climate typology is that proposed by [32] and later on modified in [33]. It is an empirical classification that divides the climate into five major types, which are represented by the capital letters A (tropical zone), B (dry zone), C (temperate zone), D (continental zone), and E (polar zone). Each of these climate types except for B is defined by temperature criteria. More recent classifications can been found in the AR6 of the IPCC (2021, 2022) but all of them share the spirit of the original one of [32].

The climate classification we propose in this section is also based on temperature data and it has three simple distinctive characteristics:

- It considers the whole temperature distribution and not only the average
- It has a dynamic long-run nature: it is based on the evolution of the trend of the temperature quantiles (lower and upper)
- It can be easily tested.

**Definition 4**. *(Warming Typology)*: *We define four types of warming processes*:

- **W0**: *There is no trend in any of the quantiles (No warming).*
- **W1**: *All the location distributional characteristics have the same positive trend (dispersion does not contain a trend)*
- **W2**: *The Lower quantiles have a larger positive trend than the Upper quantiles (dispersion has a negative trend)*
- **W3**: *The Upper quantiles have a larger positive trend than the Lower quantiles (dispersion has a positive trend).*

Climate is understood, unlike weather, as a medium and long-term phenomenon and, therefore, it is crucial to take trends into account. Notice that this typology can be used to describe macroclimate as well as microclimate locations.

The literature on global and local warming that solely examines the trend behavior of the central part of the temperature distribution (mean or median) overlooks valuable information that can be utilized to describe the entire warming process. It is possible that by analyzing only the mean temperature of a region, one may conclude that there is no significant warming (no trend in the mean), while clear warming (trend) exists in other parts of the temperature distribution. The proposed warming typology takes into account the trend behavior of the entire temperature distribution, not just the mean temperature of a region. This approach offers a more comprehensive understanding of the warming process and is valuable for characterizing and measuring climate change heterogeneity. However, this typology does not address the intensity of the warming process and its dynamics. Part of this intensity is captured in the following testable definitions of warming acceleration and warming amplification.

**Definition 5**. *(Warming Acceleration)*: *We say that there is warming acceleration in a distributional temperature characteristic C*_*t*_ *between the time periods t*_1_ = (1, …, *s*) *and t*_2_ = (*s* + 1, …, *T*) *if in the following two regressions*:

$$\begin{array}{c} C_{t}=\alpha_{1}+\beta_{1}t+u_{t},\;t=1,\ldots ,s,\ldots ,T, \end{array}$$ (5)

$$\begin{array}{c} C_{t}=\alpha_{2}+\beta_{2}t+u_{t},\;t=s+1,\ldots ,T, \end{array}$$ (6)

*the second trend slope is larger than the first one*: *β*_2_ > *β*_1_.

In practice, we implement this definition by testing in the previous system the null hypothesis *β*_2_ = *β*_1_ against the alternative *β*_2_ > *β*_1_ An alternative warming acceleration test can be formed by testing for a structural break at *t* = *s*. The preference for the approach of Definition 5 is due to its close alignment with the existing narrative on warming acceleration in the climate literature (see [34]).

The existence of acceleration can be considered an example of a non-linearity in the climate change process (see [10]). Warming acceleration is behind the recent surge in record-breaking heat. This acceleration has elevated the baseline for temperature fluctuations, leading to unprecedented spikes in warmth and contributing to extreme heat waves, wildfires, and other extreme climate events.

**Definition 6**. *(Warming Amplification with respect to the mean)*: *We say that there is a warming amplification in distributional characteristic C*_*t*_ *with respect the mean if in the following regression*:

$$\begin{array}{c} C_{t}=\alpha +\beta \;mean_{t}+\epsilon_{t} \end{array}$$ (7)

*the mean slope is greater than one*: *β* > 1.

When the mean, *mean*_*t*_, and *C*_*t*_ come from the same distribution, we name this “inner” warming amplification. If the mean originates from an external environment, it is refereed to as “outer” warming amplification. The implications of warming amplification, particularly but not only in the Arctic, are far-reaching and have significant environmental consequences like decline of sea ice cover, retreat of the Greenland ice sheet, retreat of regional glaciers, permafrost degradation, disturbances in ecosystems, influences on extreme climate events in lower latitudes, etc. Understanding theses implications is crucial for informing climate policies and adaptation strategies on both regional and global scale. And this is why is important to have a definition of amplification that can be easily tested.

Both concepts, acceleration and amplification, introduce a quantitative dimension to the ordinarily defined classification. For example, the acceleration, which has a dynamic character, allows us to observe the transition from one type of climate to another. Amplification, on the other hand, makes it possible to compare the magnitude of the trends that define each type of climate. It should be noted that, although static in nature, it can be computed recursively at different points in time.

In the previous definitions, we categorize the warming process of different regions, which is essential for designing local mitigation and adaptation policies. However, it is also important to compare the different climate change processes of two regions in order to characterize climate heterogeneity independently of the type of warming they are experiencing. For this purpose, we propose the following definition, which is akin to the stochastic dominance concept used in the economic-finance literature.

**Definition 7**. *(Warming Dominance (WD))*: *We say that the temperature distribution of **Region** A warming dominates (WD) the temperature distribution of **Region** B if in the following regression, with q*_*τ*_ *the quantile τ of temperature distributions of regions A or B*:

$$\begin{array}{c} q_{\tau t}\left(A\right)-q_{\tau t}\left(B\right)=\alpha_{\tau }+\beta_{\tau }t+u_{\tau t}, \end{array}$$ (8)

*β*_*τ*_ ≥ 0 *for all* 0 < *τ* < 1 and there is at least one value τ* *for which a strict inequality holds*.

It is also possible to have only *partial* *WD*. For instance, in the lower or upper quantiles. The concept of *WD* is analyzed in full detail in [35].

## 3 The data

### 3.1 The Globe

For the Globe, we utilize the Climate Research Unit (CRU) database, which provides monthly and yearly land and sea temperature data for both hemispheres from 1850 to the present, collected from different stations around the world. A recent revision of the methodology can be found in [36]. Each station temperature is converted to an anomaly, taking 1961–1990 as the base period, and each grid-box value, on a five-degree grid, is the mean of all the station anomalies within that grid box. This database (in particular, the annual temperature of the Northern Hemisphere) has become one of the most widely used to illustrate GW from records of thermometer readings. These records form the blade of the well-known “hockey stick” graph, frequently used by academics and other institutions, such as, the IPCC. In this paper, we prefer to base our analysis on raw station data, as in [4]. Similar results are obtained with stable grid anomaly data. The crucial issue in dealing with temperature data is the aggregation process. Failing to maintain consistent stations or grids across the entire sample increases the likelihood of encountering outliers (see [37] for a method to evaluate their impact on climate data) and potentially spurious unit roots in distributional characteristics such as average temperature (see [38]).

**Fig 1:**
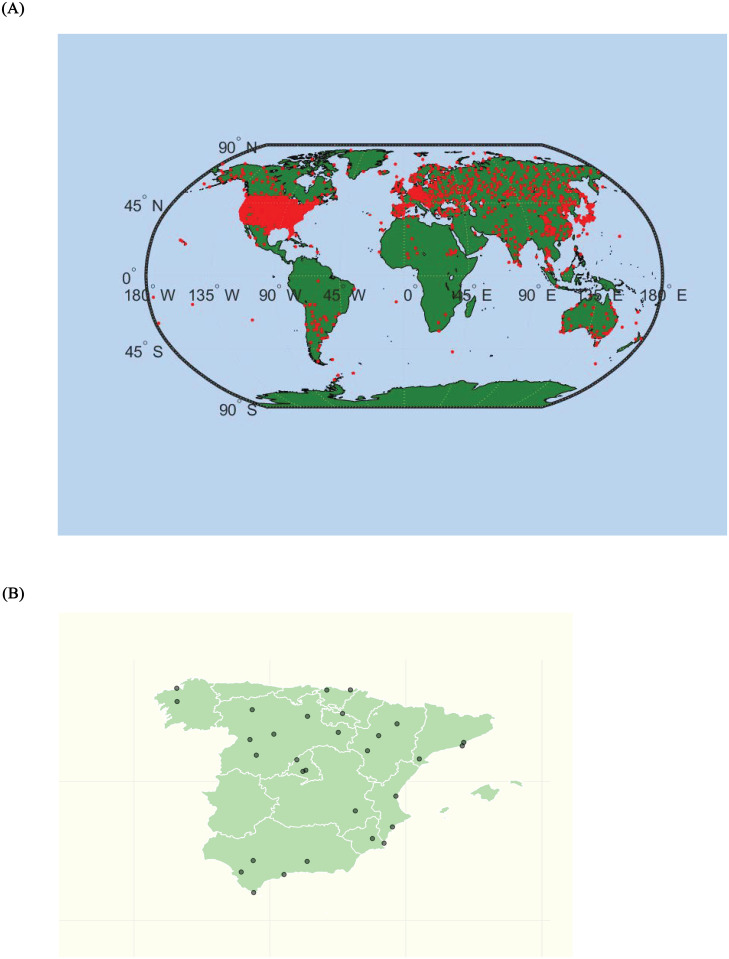
Geographical distribution of stations. A: The Globe. Selected stations, CRU data 1950–2019 B: Spain. Selected stations, AEMET data 1950–2019. *Sources*: A: Own elaboration based on CRU data with the toolbox Mapping under Matlab license version 2022b; B: Own elaboration with R and data from IGN and AEMET; the geographic data of the base map are obtained from the free-use tool developed by the National Geographic Institute of Spain and can be consulted at: https://comunidadsig.maps.arcgis.com/home/item.html?id=e75892d1a49646d8a29705ac6680f981.

The database provides data from 1850 to nowadays, although due to the high variability at the beginning of the period it is customary in the literature to begin in 1880. In this work, we have selected the stations that are permanently present in the period 1950–2019 according to the concept of the station-month unit. In this way, the results are comparable with those obtained for the benchmark country Spain. Notice that this period corresponds to the Anthropocene period of time. Although there are 10,633 stations on record, the effective number fluctuates each year and there are only 2,192 stations with data for all the years in the sample period, which yields 19,284 station-month units each year (see this geographical distribution in the map in Fig 1. In the CRU data there are 115 Spanish stations. However, after removing stations not present for the whole 1880 to 2019 period, only Madrid-Retiro, Valladolid and Soria remain. Since 1950, applying the same criteria, only 30 remain. In summary, we analyze raw land temperature observational global data (stations instead of grids) for the period 1950 to 2019, compute station-month units that remain all the time and with these build the annual distributional characteristics.

### 3.2 Spain

The measurement of meteorological information in Spain started in the eighteenth century. However, it was not until the mid-nineteenth century that reliable and regular data became available. In Spain, there are four main sources of meteorological information: the Resumen Anual, Boletín Diario, Boletín Mensual de Climatología and Calendario Meteorológico. These were first published in 1866, 1893, 1940 and 1943, respectively. A detailed explanation of the different sources can be found in [39].

Currently, AEMET (Agencia Estatal de Meterología) is the agency responsible for storing, managing and providing meteorological data to the public. Some of the historical publications, such as the Boletín Diario and Calendario Meteorológico can be found in digital format in their respective archives for whose use it is necessary to use some kind of Optical Character Recognition (OCR) software.

In 2015, AEMET developed AEMET OpenData, an Application Programming Interface (API REST) that allows the dissemination and reuse of Spanish meteorological and climatological information. To use it, the user needs to obtain an API key to allow access to the application. Then, either through the GUI or through a programming language such as Java or Python, the user can request data. More information about the use of the API and the resolution about the use of the data can be found on their webpage.

In this paper, we are concerned with Spanish daily station data, specifically temperature data. Each station records the minimum, maximum and average temperature as well as the amount of precipitation, measured as liters per square meter. The data period ranges from 1920 to 2019. However, in 1920 there were only 13 provinces (out of 52) who had stations available. It was not until 1965 that all the 52 provinces had at least one working meteorological station. Moreover, it is important to keep in mind that the number of stations has increased substantially from only 14 stations in 1920 to more than 250 in 2019. With this information in mind, we select the longest span of time that guarantees a wide sample of stations so that all the geographical areas of peninsular Spain are represented. For this reason, we decided to work with station data from 1950 to 2019. There are 30 stations whose geographical distribution is displayed in the map in Fig 1. The original daily data are converted into monthly data, so that we finally work with a total of 30x12 station-month units corresponding to peninsular Spain and, consequently, we have 360 observations each year with which to construct the annual distributional characteristics.

Table 1 contains a summary of the data detail for the Globe and Spain.

**Table 1:** Data details.

	The Globe	Spain
Source	CRUTEM version 5.0.1.0	AEMET
Sample of the data	1850–2023	1920–2023
Period analyzed	1950–2019	1950–2019
Frequency	Monthly	Daily
Number of total stations	10,633	292
Number of effective stations	2,192	30
(maintained during the whole period)		
Total of month-station units	19,284	360

## 4 Empirical strategy

In this section we apply our three-step quantitative methodology to show the existent climate heterogeneity between the Globe and Spain as well as within Spain, between Madrid and Barcelona. Because all our definitions are written in a testable regression format, it is straightforward to empirically apply them. First, we test for the existence of warming by testing the existence of a trend in a given distributional characteristic. How common are the trends of the different characteristics (revealed by a co-trending test) determine the warming typology. Second, the strength of the warming process is tested by testing the hypothesis of warming acceleration and warming amplification. And third, independently of the warming typology, we determine how the warming process of Spain compares with that of the Globe as a whole (we do the same for Madrid and Barcelona). This is done by testing for warming dominance.

The results are presented according to the following steps: first, we apply our trend test (see Definition 2) to determine the existence of global or local warming and test for any possible warming acceleration; second, we test different co-trending hypotheses to determine the type of warming of each area; thirdly, we test the warming amplification hypothesis for different quantiles with respect to the mean (of the Globe as well as of Spain): *H*_0_ : *β*_1_ = 1 versus *H*_1_ : *β*_1_ > 1 in (7); and finally, we compare the CC of different regions, for the Globe and Spain, and within Spain, between Madrid and Barcelona, with our warming dominance test (see 8). Before testing for the presence of trends in the distributional characteristics of the data, we test for the existence of unit roots. To do so, we use the well-known Augmented Dickey-Fuller test (ADF; [40]) for each characteristic *C*_*t*_:

$$\begin{array}{c} △C_{t}=\alpha +\beta C_{t-1}+\sum_{i=1}^{p}\gamma_{i}△C_{t-i}+\epsilon_{t} \end{array}$$

where *H*_0_ : *β* = 0, *H*1 : *β* < 0 and the number of lags *p* is selected by the SBIC criterion. The results, available from the authors on request, show that the null hypothesis of a unit root is rejected for all the characteristics considered.

### 4.1 Global warming: The Globe

**Fig 2:**
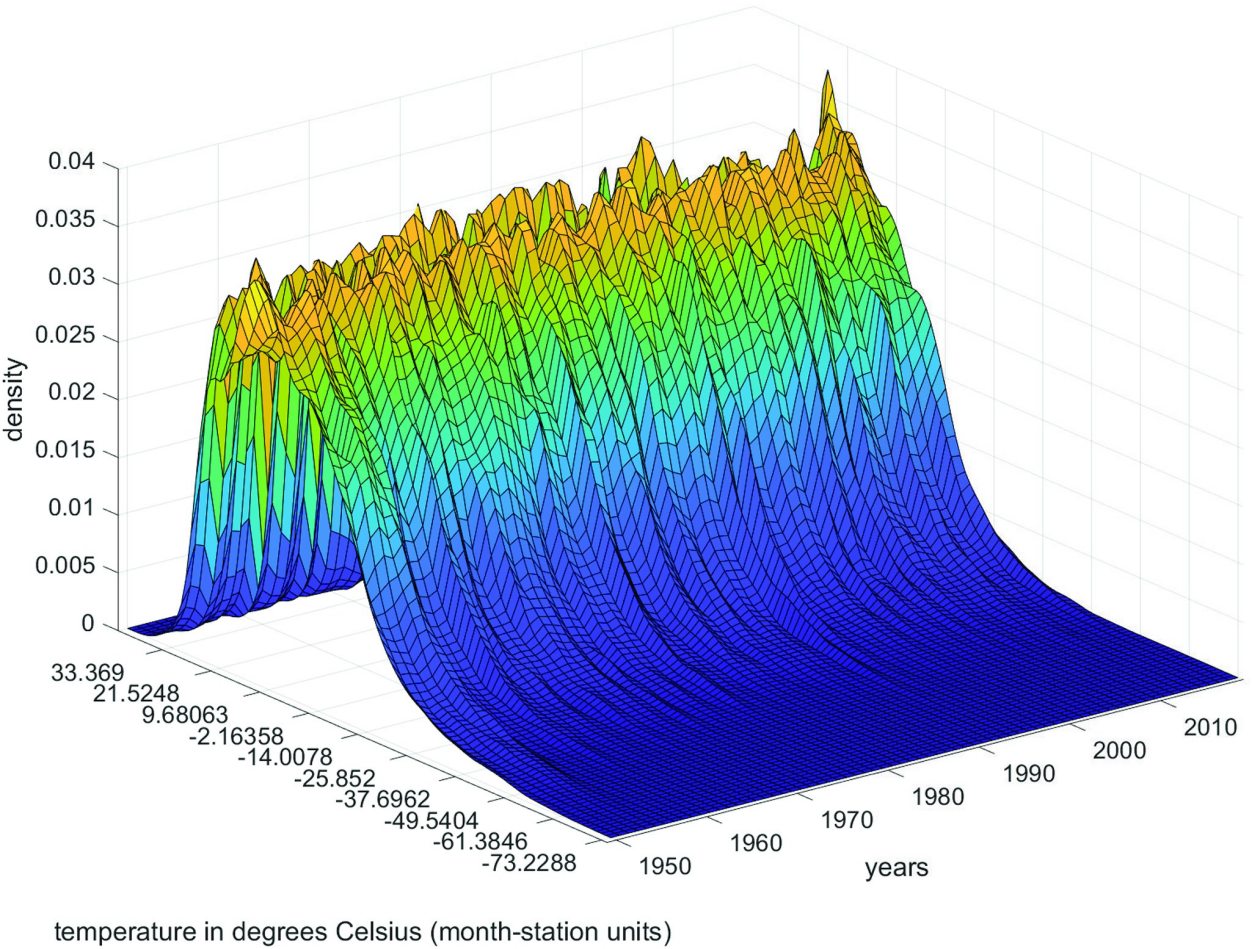
Global annual temperature density calculated with monthly data across stations.

**Fig 3:**
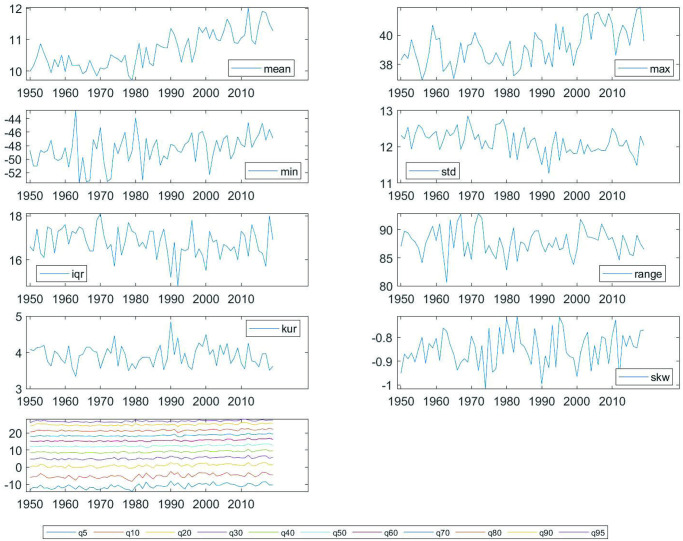
Characteristics of temperature data in the Globe (monthly data across stations, CRU, 1950–2019).

**Fig 4:**
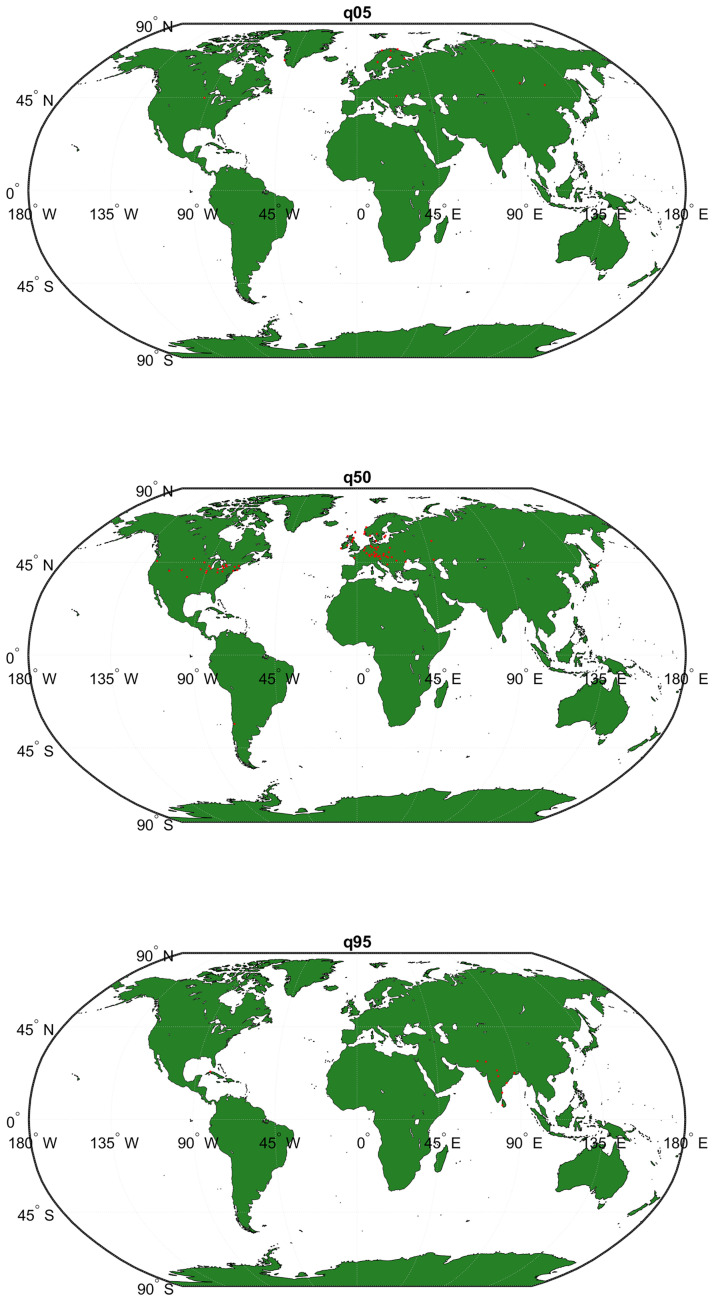
Geographical location by quantiles (monthly data across stations, CRU, 1950–2019). *Sources*: Own elaboration based on CRU data with the toolbox Mapping under Matlab license version 2022b.

The cross-sectional analysis is approached under two assumptions. First, choosing a sufficiently long and representative period of the geographical diversity of the Globe and the Spanish Iberian Peninsula, 1950–2019. Second, we work with month-station units from monthly observations to construct the annual observations of the time series object from the data supplied by the stations, following a methodology similar to that carried out for the whole planet in [21]. The decision to work with monthly data instead of daily in the cross-sectional approach has been based on its compatibility with the data available for the Globe. The results with daily averages for Spain are very similar. The study comprises the steps described in the previous section. Figs 2 and 3 show the time evolution of the Global temperature densities and their different distributional characteristics from 1950 to 2019. Importance to notice that the unconditional quantiles correspond to locations-latitudes (Fig 4). This is a simple way of incorporating the spatial dimension into the analysis. In some sense, to go from analyzing the average temperature to analyze all the quantiles is like going from a zero dimension energy balance climate model to a one dimensional model. The data in the three figures are obtained from stations that report data throughout the sample period.

Table 2 shows a positive trend in the mean as well as in all the quantiles. This indicates the clear existence of Global warming, more pronounced (larger trend) in the lower part of the distribution (a negative trend in the dispersion measures). There is a lack of consensus regarding the acceleration of the warming process. Some argue that warming is steadily increasing but not accelerating, while others, such as [34], assert a clear acceleration due to the effect of clean air regulations that have reduced aerosol pollution and the amount of sulfur in fuel used by ocean shipping. Our quantitative findings align with the latter perspective, indicating a clear acceleration in the warming process in the mean and in all the quantiles above *q30*.

**Table 2:** Trend acceleration hypothesis (CRU monthly data across stations, 1950–2019).

names/periods	Trend test by periods		Acceleration test
	1950–2019	1970–2019	1950–2019, 1970–2019
mean	0.0213	0.0300	2.2023
	(0.0000)	(0.0000)	(0.0147)
max	0.0361	0.0523	1.1217
	(0.0000)	(0.0001)	(0.1320)
min	0.0423	-0.0109	0.5016
	(0.0000)	(0.5867)	(0.3084)
std	-0.0070	-0.0057	0.1776
	(0.0000)	(0.0570)	(0.4296)
iqr	-0.0067	-0.0043	0.2454
	(0.0435)	(0.4183)	(0.4033)
rank	-0.0062	0.0632	0.2181
	(0.5876)	(0.0005)	(0.4138)
kur	-0.0010	0.0001	0.0445
	(0.5205)	(0.9566)	(0.4823)
skw	0.0006	0.0003	0.0301
	(0.0577)	(0.5726)	(0.4880)
q05	0.0404	0.0468	0.7035
	(0.0000)	(0.0000)	(0.2415)
q10	0.0305	0.0406	0.9273
	(0.0000)	(0.0001)	(0.1777)
q20	0.0253	0.0342	1.0156
	(0.0000)	(0.0000)	(0.1558)
q30	0.0215	0.0280	1.2056
	(0.0000)	(0.0000)	(0.1150)
q40	0.0192	0.0293	1.9873
	(0.0000)	(0.0000)	(0.0245)
q50	0.0179	0.0268	1.8614
	(0.0000)	(0.0000)	(0.0324)
q60	0.0185	0.0291	2.1971
	(0.0000)	(0.0000)	(0.0149)
q70	0.0185	0.0288	2.5770
	(0.0000)	(0.0000)	(0.0055)
q80	0.0160	0.0257	2.2460
	(0.0000)	(0.0000)	(0.0132)
q90	0.0146	0.0243	2.0848
	(0.0005)	(0.0000)	(0.0195)
q95	0.0143	0.0239	1.7520
	(0.0001)	(0.0000)	(0.0410)

*Notes*: OLS estimates and HAC p-values in parenthesis of the *t*_*β* = 0_ test from regression: *C*_*t*_ = *α* + *βt* + *u*_*t*_, for two different time periods. For the acceleration hypothesis we run the system: *C*_*t*_ = *α*_1_ + *β*_1_*t* + *u*_*t*_, *t* = 1, …, *s*, …, *T*, *C*_*t*_ = *α*_2_ + *β*_2_*t* + *u*_*t*_, *t* = *s* + 1, …, *T*, and test the null hypothesis *β*_2_ = *β*_1_ against the alternative *β*_2_ > *β*_1_. We show the value of the t-statistic and its HAC p-value.

From the co-trending analysis (see Tables 3 and 4) we can determine the type of warming process characterizing the whole Globe. Table 3 indicates that in the period 1950–2019 the Globe experimented a *W2* warming type (the lower part of the temperature distribution grows faster than the middle and upper part, implying *iqr* and *std* have a negative trend). Similar results are maintained for the period 1970–2019 (in this case only the dispersion measure *std* has a negative trend).

**Table 3:** Co-trending analysis (CRU montly data, 1950–2019).

Joint hypothesis tests	Wald test	p-value
All quantiles (q05, q10,…,q90, q95)	25.143	0.005
Lower quantiles (q05, q10, q20, q30)	9.545	0.023
Medium quantiles (q40, q50, q60)	0.078	0.962
Upper quantiles (q70, q80, q90, q95)	1.099	0.777
Lower-Medium quantiles (q05, q10, q20, q30, q40, q50, q60)	17.691	0.007
Medium-Upper quantiles (q40, q50, q60, q70, q80, q90, q95)	2.041	0.916
Lower-Upper quantiles (q05, q10, q20,q30, q70, q80, q90, q95)	24.683	0.001
Spacing hypothesis	Trend-coeff.	p-value
q50-q05	-0.022	0.000
q95-q50	-0.004	0.193
q95-q05	-0.026	0.000
q75-q25 (iqr)	-0.007	0.043

*Notes*: Annual distributional characteristics (quantiles) of temperature. The top panel shows the Wald test of the null hypothesis of equality of trend coefficients for a given set of characteristics. In the bottom panel, the TT is applied to the difference between two representative quantiles.

**Table 4:** Co-trending analysis (CRU montly data, 1970–2019).

Joint hypothesis tests	Wald test	p-value
All quantiles (q05, q10,…,q90, q95)	18.478	0.047
Lower quantiles (q05, q10, q20, q30)	5.523	0.137
Medium quantiles (q40, q50, q60)	0.569	0.752
Upper quantiles (q70, q80, q90, q95)	2.667	0.446
Lower-Medium quantiles (q05, q10, q20, q30, q40, q50, q60)	7.606	0.268
Medium-Upper quantiles (q40, q50, q60, q70, q80, q90, q95)	6.714	0.348
Lower-Upper quantiles (q05, q10, q20,q30, q70, q80, q90, q95)	14.520	0.043
Spacing hypothesis	Trend-coeff.	p-value
q50-q05	-0.020	0.047
q95-q50	-0.003	0.462
q95-q05	-0.023	0.048
q75-q25 (iqr)	-0.004	0.418

*Notes*: Annual distributional characteristics (quantiles) of temperature. The top panel shows the Wald test of the null hypothesis of equality of trend coefficients for a given set of characteristics. In the bottom panel, the TT is applied to the difference between two representative quantiles.

The asymmetric amplification results shown in Table 5 reinforce the *W2* typology for the whole Globe: an increase of one degree in the global mean temperature increases the lower quantiles by more than one degree. In particular, about the Arctic Amplification (AA) (see [41] for a survey of this concept), we obtain (see Table 5) that the Arctic (*q05*) has warmed twice the global average. In the literature, there is little consensus on the magnitude of the recent AA. Numerous recent studies report the Arctic having warmed either almost twice [42], about twice [43], or more than twice [44, 45] as fast as the global average. Our findings also indicate that the amplification of warming extends beyond the Arctic, reaching lower latitude locations (*q10*, *q20*, and *q30*). They also demonstrate that Arctic warming amplification has peaked, with the amplification being larger for the period 1950–2019 than for 1970–2019. This is consistent with previous research (see [46, 47]).

**Table 5:** Amplification hypotheses (CRU monthly data across stations, 1950–2019).

periods/variables	1950–2019	1970–2019
q05	2.00	1.83
	(0.000)	(0.000)
q10	1.79	1.73
	(0.000)	(0.001)
q20	1.41	1.37
	(0.000)	(0.000)
q30	1.07	1.00
	(0.089)	(0.502)
q40	0.88	0.91
	(0.999)	(0.973)
q50	0.74	0.81
	(1.000)	(0.997)
q60	0.74	0.85
	(0.999)	(0.973)
q70	0.77	0.85
	(1.000)	(0.988)
q80	0.72	0.78
	(1.000)	(1.000)
q90	0.69	0.70
	(1.000)	(1.000)
q95	0.60	0.64
	(1.000)	(1.000)

*Notes*: OLS estimates and HAC p-values of the t-statistic of testing *H*_0_ : *β*_*i*_ = 1 versus *H*_1_ : *β*_*i*_ > 1 in the regression: *C*_*it*_ = *α*_*i*_ + *β*_*i*_ *mean*_*t*_ + *ϵ*_*it*_. *mean* refers to the average of the Global temperature distribution.

In summary, the results from our various proposed tests for the evolution of the trend of the entire temperature distribution suggest that the Globe can be classified as undergoing a type *W2* warming process. This type of warming may have more significant consequences for ice melting, sea level increases, permafrost, migrations, etc., compared to other warming types.

### 4.2 Local warming: Spain

**Fig 5:**
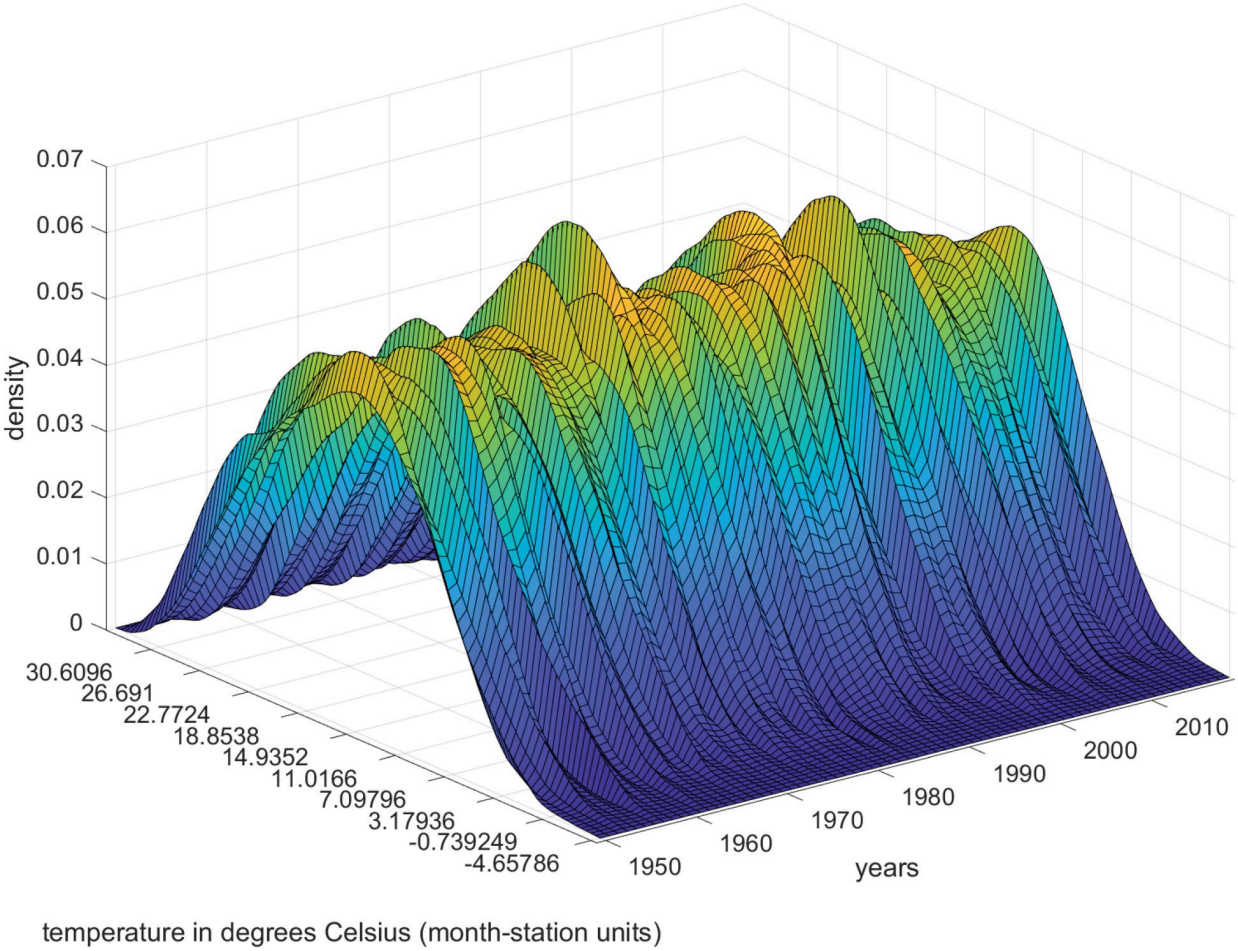
Spain annual temperature density calculated with monthly data across stations.

**Fig 6:**
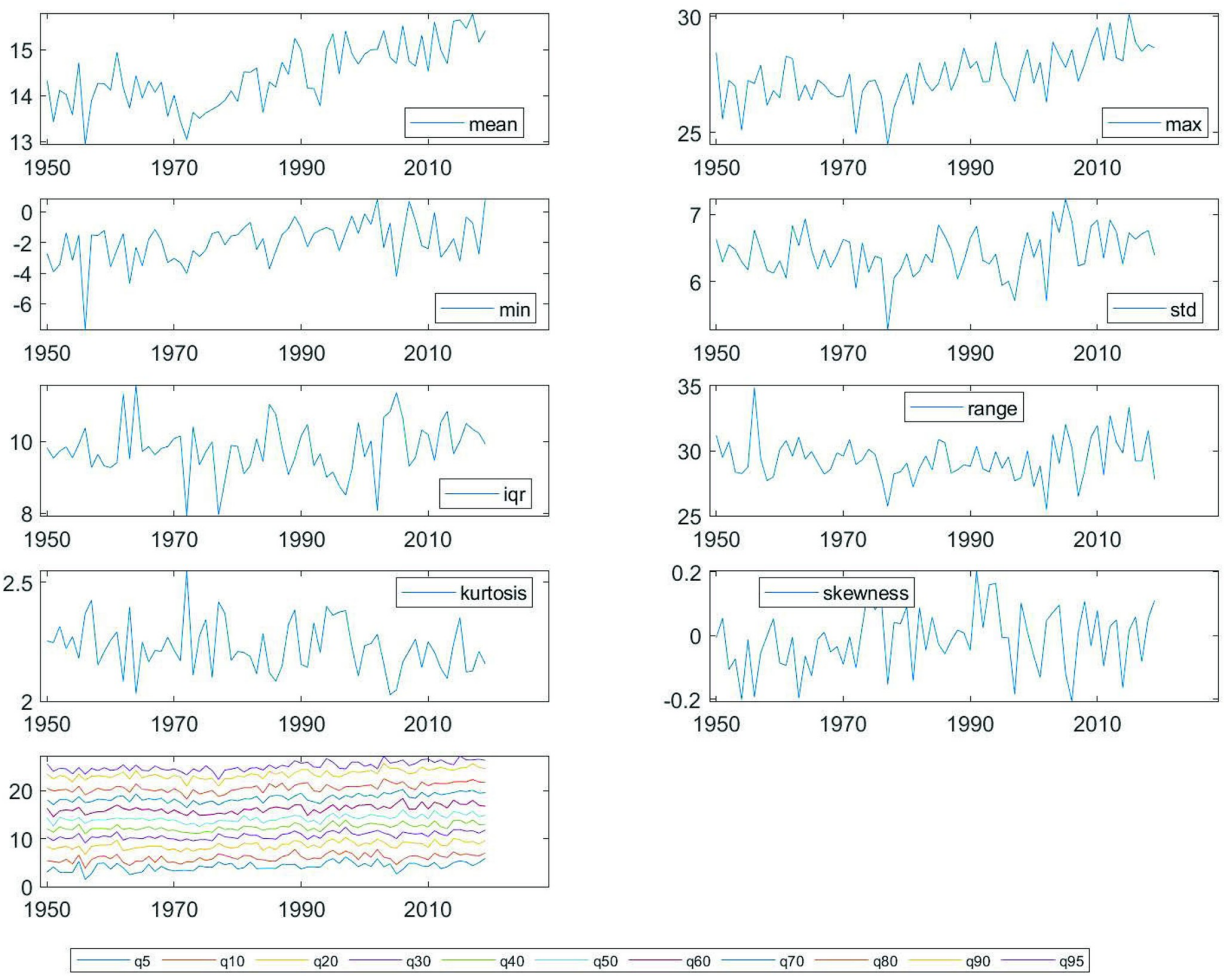
Characteristics of temperature data in Spain with stations selected since 1950 (monthly data across stations, AEMET, 1950–2019).

In this section, we carry out a similar analysis to that described in the previous subsection for the Globe. The density of the data and the evolution of characteristics are displayed, respectively in Figs 5 and 6.

We find positive and significant trends in the *mean*, *max*, *min* and all the quantiles. Therefore from definition 1, we conclude there exists a clear local warming (see Table 6).

**Table 6:** Trend acceleration hypothesis (Spain monthly data across stations, AEMET, 1950–2019).

names/periods	Trend test by periods		Acceleration test
	1950–2019	1970–2019	1950–2019, 1970–2019
mean	0.0242	0.0389	3.0294
	(0.0000)	(0.0000)	(0.0015)
max	0.0312	0.0526	2.7871
	(0.0000)	(0.0000)	(0.0030)
min	0.0289	0.0251	-0.2557
	(0.0000)	(0.0654)	(0.6007)
std	0.0036	0.0098	1.7952
	(0.0518)	(0.0021)	(0.0374)
iqr	0.0051	0.0158	1.8197
	(0.1793)	(0.0028)	(0.0355)
rank	0.0023	0.0276	1.2705
	(0.8249)	(0.1127)	(0.1030)
kur	-0.0010	-0.0018	-0.9191
	(0.0203)	(0.0198)	(0.8202)
skw	0.0011	-0.0002	-1.5989
	(0.0271)	(0.7423)	(0.9439)
q05	0.0227	0.0206	-0.2559
	(0.0000)	(0.0059)	(0.6008)
q10	0.0200	0.0203	0.0406
	(0.0000)	(0.0077)	(0.4838)
q20	0.0209	0.0300	1.4158
	(0.0000)	(0.0000)	(0.0796)
q30	0.0221	0.0333	2.0100
	(0.0000)	(0.0000)	(0.0232)
q40	0.0213	0.0366	2.4867
	(0.0000)	(0.0000)	(0.0071)
q50	0.0211	0.0404	3.2496
	(0.0000)	(0.0000)	(0.0007)
q60	0.0246	0.0446	3.1147
	(0.0000)	(0.0000)	(0.0011)
q70	0.0273	0.0478	3.3143
	(0.0000)	(0.0000)	(0.0006)
q80	0.0275	0.0471	2.6949
	(0.0000)	(0.0000)	(0.0040)
q90	0.0321	0.0548	3.2441
	(0.0000)	(0.0000)	(0.0007)
q95	0.0335	0.0526	3.3568
	(0.0000)	(0.0000)	(0.0005)

*Notes*: OLS estimates and HAC p-values in parenthesis of the *t*_*β* = 0_ test from regression: *C*_*t*_ = *α* + *βt* + *u*_*t*_, for two different time periods. For the acceleration hypothesis we run the system: *C*_*t*_ = *α*_1_ + *β*_1_*t* + *u*_*t*_, *t* = 1, …, *s*, …, *T*, *C*_*t*_ = *α*_2_ + *β*_2_*t* + *u*_*t*_, *t* = *s* + 1, …, *T*, and test the null hypothesis *β*_2_ = *β*_1_ against the alternative *β*_2_ > *β*_1_. We show the value of the t-statistic and its HAC p-value.

The recursive evolution for the periods 1950–2019 and 1970–2019 shows a clear increase in the trends of the *mean*, some dispersion measures and higher quantiles (see the last column of Table 6). More precisely, there is a significant trend acceleration in most of the distributional characteristics except the lower quantiles (below *q20*). These quantiles, *q05* and *q10*, remain stable. The acceleration in Spain is reported to be stronger than the one experienced by the Globe. This indicates a notable change in the temperature distribution, particularly in the higher quantiles, which can have implications for local climate policies and environmental impacts.

The co-trending tests for the full sample 1950–2019 show a similar evolution of the trend for all the quantiles with a constant *iqr* (see Table 7). This indicates that in this period the warming process of Spain can be considered a *W1* type. More recently, 1970–2019, the co-trending tests (see Table 8) indicate the upper quantiles grow faster than the lower ones. This, together with a positive trend in the dispersion measured by the *iqr* shows that Spain has evolved from a *W1* to a *W3* warming type process.

**Table 7:** Co-trending analysis (Spain monthly data across stations, AEMET, 1950–2019).

Joint hypothesis tests	Wald test	p-value
All quantiles (q05, q10,…,q90, q95)	13.235	0.211
Lower quantiles (q05, q10, q20, q30)	0.310	0.958
Medium quantiles (q40, q50, q60)	0.438	0.803
Upper quantiles (q70, q80, q90, q95)	1.515	0.679
Lower-Medium quantiles (q05, q10, q20, q30, q40, q50, q60)	0.771	0.993
Medium-Upper quantiles (q40, q50, q60, q70, q80, q90, q95)	8.331	0.215
Lower-Upper quantiles (q05, q10, q20,q30, q70, q80, q90, q95)	11.705	0.111
Spacing hypothesis	Trend-coeff.	p-value
q50-q05	-0.002	0.786
q95-q50	0.012	0.000
q95-q05	0.011	0.096
q75-q25 (iqr)	0.005	0.179

*Notes*: Annual distributional characteristics (quantiles) of temperature. The top panel shows the Wald test of the null hypothesis of equality of trend coefficients for a given set of characteristics. In the bottom panel, the TT is applied to the difference between two representative quantiles.

**Table 8:** Co-trending analysis (Spain monthly data across stations, AEMET, 1970–2019).

Joint hypothesis tests	Wald test	p-value
All quantiles (q05, q10,…,q90, q95)	38.879	0.000
Lower quantiles (q05, q10, q20, q30)	3.121	0.373
Medium quantiles (q40, q50, q60)	1.314	0.518
Upper quantiles (q70, q80, q90, q95)	1.719	0.633
Lower-Medium quantiles (q05, q10, q20, q30, q40, q50, q60)	12.771	0.047
Medium-Upper quantiles (q40, q50, q60, q70, q80, q90, q95)	10.675	0.099
Lower-Upper quantiles (q05, q10, q20,q30, q70, q80, q90, q95)	37.892	0.000
Spacing hypothesis	Trend-coeff.	p-value
q50-q05	0.020	0.029
q95-q50	0.012	0.050
q55-q05	0.032	0.002
q75-q25 (iqr)	0.016	0.003

*Notes*: Annual distributional characteristics (quantiles) of temperature. The top panel shows the Wald test of the null hypothesis of equality of trend coefficients for a given set of characteristics. In the bottom panel, the TT is applied to the difference between two representative quantiles.

Finally, no evidence of “inner” amplification during the period 1950–2019 is found in the lower quantiles. Regarding the upper quantiles, we found both “inner” and “outer” amplification in the second period, which supports the previous finding of a transition from type *W1* to type *W3* (see Table 9). With respect the “outer” amplification it is important to note that while there is no amplification with respect to the average global temperature in the median, there is amplification in the upper quantiles (approximately 1.4^*o*^*C*) This is evidenced by the heatwaves experienced in the country (see [48]).

**Table 9:** Amplification hypothesis (Spain monthly data, AEMET 1950–2019).

periods/variables	1950–2019	1970–2019	1950–2019	1970–2019
	Inner		Outer	
q05	0.80	0.56	0.55	0.39
	(0.866)	(0.998)	(0.990)	(0.996)
q10	0.83	0.65	0.62	0.52
	(0.899)	(0.994)	(0.992)	(0.986)
q20	0.94	0.90	0.76	0.81
	(0.816)	(0.890)	(0.993)	(0.899)
q30	0.93	0.91	0.77	0.87
	(0.935)	(0.929)	(0.997)	(0.834)
q40	0.97	1.03	0.80	0.97
	(0.744)	(0.318)	(0.978)	(0.566)
q50	0.98	1.10	0.83	1.12
	(0.612)	(0.067)	(0.944)	(0.212)
q60	1.09	1.15	0.96	1.23
	(0.103)	(0.051)	(0.619)	(0.056)
q70	1.11	1.16	1.05	1.30
	(0.040)	(0.006)	(0.350)	(0.028)
q80	1.11	1.14	1.06	1.29
	(0.083)	(0.071)	(0.325)	(0.060)
q90	1.14	1.16	1.19	1.45
	(0.101)	(0.118)	(0.078)	(0.007)
q95	1.10	1.09	1.18	1.36
	(0.089)	(0.191)	(0.051)	(0.008)

*Notes*: OLS estimates and HAC p-values of the t-statistic of testing *H*_0_ : *β* = 1 versus *H*_1_ : *β* > 1 in the regression: *C*_*t*_ = *α* + *β* *mean*_*t*_ + *ϵ*_*t*_. *mean* refers to the average of the Spanish/Global temperature distribution for the “inner” and “outer”cases, respectively.

Summing up, with our proposed tests for the evolution of the trend of the whole temperature distribution, we conclude that Spain has evolved from a *W1* type to a much more dangerous *W3* type. The results of acceleration and dynamic amplification reinforce the finding of this transition to type *W3*.

### 4.3 Micro-local warming: Madrid and Barcelona

The existence of warming heterogeneity implies that in order to design more efficient mitigation policies, they have to be developed at different levels: global, country, region etc. How local we need to go will depend on the existing degree of micro-warming heterogeneity. In this subsection, we go to the smallest level, climate station level. We analyze, within Spain, the warming process in two meteorological stations corresponding to two cities: Madrid (Retiro park station) and Barcelona (Fabra station). From Madrid and Barcelona there is data since 1920’s, nevertheless we began the study in 1950 for consistency with the previous analysis of Spain and the Globe. Obviously, the data provided by these stations is not cross-sectional data but directly pure time series data. Our methodology can be easily applied to higher frequency time series, in this case daily data, to compute the distributional characteristics (see Fig A1 and Fig A2 in S1 Appendix). See the application to Central England in GG2020 and in [49] to Madrid, Zaragoza and Oxford.

The results are shown in the S1 Appendix. These two stations, Madrid-Retiro and Barcelona-Fabra clearly experience two different types of warming. First, there is evidence of micro-local warming, understood as the presence of significant and positive trends, in all the important temperature distributional characteristics of both stations. The acceleration phenomenon is also clearly detected, in other words, the warming increases as time passes (see Tables A1 and A5 in S1 Appendix). Secondly, from the co-trending tests (Tables A2, A3, A6 and A7 in S1 Appendix), it can be concluded that the warming process of Madrid-Retiro is type *W3* while for Barcelona-Fabra it is type *W1*. In both cases the warming typology is stable through both sample periods (1950–2019 and 1970–2019). Thirdly, as expected, Madrid-Retiro presents “inner” and “outer” amplification for the upper quantiles, while Barcelona-Fabra does so only for the center part of its temperature distribution (see Tables A4 and A8 in S1 Appendix).

In summary, there is clear evidence of heterogeneous warming within Spain. While Madrid, with a Cold Semi-Arid climate according to the Köppenn-Geiger classification [32, 33], exhibits a similar warming pattern to that of peninsular Spain (1970–2019) *W3*, Barcelona, characterized by a Mediterranean coastline climate, maintains a *W1* typology. These distinct warming processes may necessitate different mitigation and adaptation policies at both the national and local levels. The differences in climate classifications based on associated warming trends highlight the need for tailored approaches to address the specific challenges posed by climate change in different regions of Spain.

## 5 Comparing warming results

**Fig 7:**
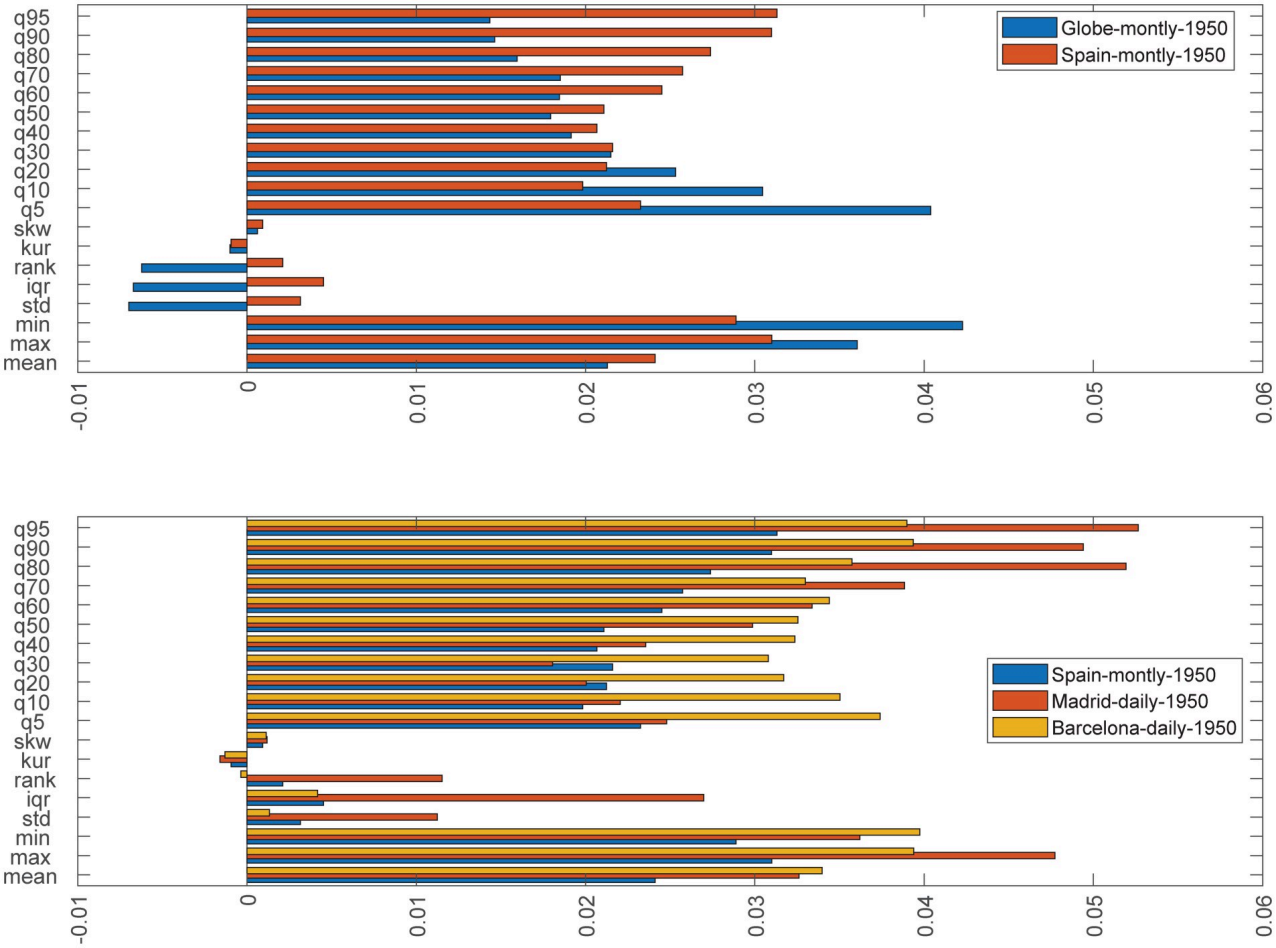
Trend evolution of different temperature distributional characteristics. *Notes*: The bars represent the intensity of the trends found in each characteristic measured through the value of the *β*-coefficient estimated in the regression *C*_*t*_ = *α* + *βt* + *u*_*t*_.

In the previous three subsections, we have used the first two steps of our three-step methodology to quantitatively describe, in a testable format, the warming process of the Globe, Spain, Madrid and Barcelona. A summary of these results is presented in Table 10 and Fig 7. Finding the possible causes behind the warming types *W1*, *W2*, and *W3*, is beyond the scope of this paper and will be object of future research. Some ideas from the literature, on diurnal temperature asymmetry (Diurnal Temperature Range = *DTR* = *T*_*max*_ − *T*_*min*_) suggest as possible causes for *W2* the cloud coverage [50] and the planetary boundary layer (see [51]). For *W3*, the process of desertification (see [50]).

**Table 10:** Summary of results.

Cross analysis						
Sample	Period	Type	Acceleration	Amplification		Dominance
				Inner	Outer	
The Globe						
	1950–2019	*W2*	[*mean* *q40,…, q95]*	[*q05,…, q30]*		[*q05]*
	1970–2019	*W2*		[*q05,…, q20]*		
Spain						
	1950–2019	*W1*	[*mean, std, iqr, rank, q20,…, q95]*	[*q70, q80, q95]*	[*q90, q95]*	[q60,…, q95]
	1970–2019	*W3*		[*q50,…, q80]*	[*q60,…, q95]*	
Time analysis						
Sample	Period	Type	Acceleration	Amplification		Dominance
Madrid, Retiro Station						
	1950–2019	*W3*	[*mean, std, rank,* *q40, …, q95]*	[*q50,…, q95]*	[*q40,…, q95]*	[q80,…, q95]
	1970–2019	*W3*		[*q50,…, q95]*	[*q40,…, q95]*	
Barcelona, Fabra Station						
	1950–2019	*W1*	[*mean,* *q20,…, q95]*	-	[*q30,…, q90]*	*[q05,…, q40]*
	1970–2019	*W1*		[*q60, q70]*	[*q30,…, q70]*	

*Notes*: For Spain and the Globe we build characteristics from station-months units. For Madrid and Barcelona we use daily frequency time series. A significance level of 10% is considered for all tests and characteristics.

**Fig 8:**
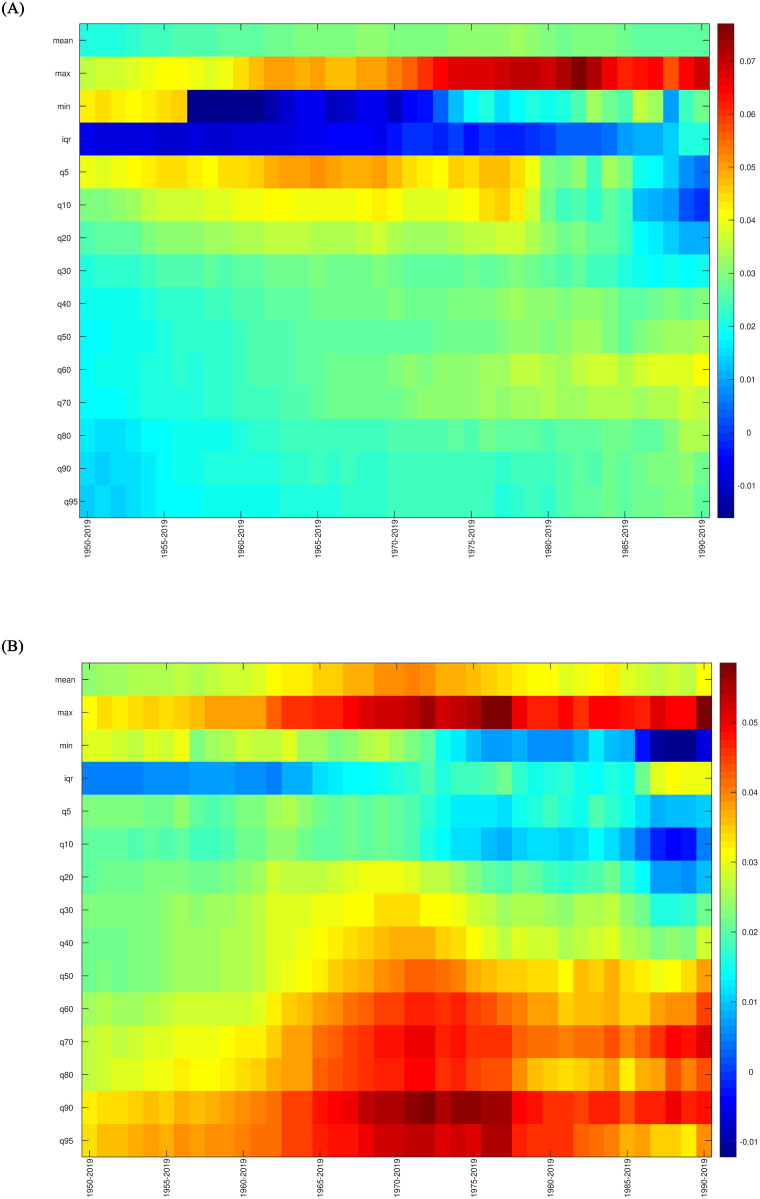
Comparing heatmaps. A: Spain. B: The Globe. *Notes*: The color scale on the right side of the figure shows the intensity of the trend, based on the value of the *β*-coefficient estimated in the regression *C*_*t*_ = *α* + *βt* + *u*_*t*_.

In this section, we demonstrate the presence of climate change heterogeneity by comparing the warming process of the examined regions. This is achieved through the use of the warming dominance test, which constitutes our third step. The numerical outcomes of the test are detailed in Table 11. Our findings reveal that the warming in Spain surpasses that of the Globe in the upper quantiles, while it is exceeded in the lower tail of the distribution corresponding to the Arctic region (see the details of the evolution in the heatmaps of Fig 8). These results, based on the entire distribution, substantiate the notion, as upheld by European institutions and reflected in international reports, of the greater intensity of climate change in the Iberian Peninsula. Furthermore, the warming in Madrid dominates that of Barcelona in the upper quantiles, whereas the opposite is observed in the lower quantiles. This latter outcome aligns with the concept that regions in proximity to the sea experience milder upper temperatures. It is important to note that when only the median (or the mean) is considered, as is typical in most standard literature, there is no warming dominance between Spain and the Globe or between Madrid and Barcelona.

**Table 11:** Warming dominance.

	Spain-Globe		Madrid-Barcelona	
Quantile	*β*	t-ratio	*β*	t-ratio
q05	-0.018	(-2.770)	-0.013	(-3.730)
q10	-0.010	(-1.504)	-0.013	(-4.215)
q20	-0.004	(-0.950)	-0.012	(-2.988)
q30	0.001	(0.180)	-0.013	(-4.164)
q40	0.002	(0.788)	-0.009	(-2.909)
q50	0.003	(1.025)	-0.003	(-0.701)
q60	0.006	(1.933)	-0.001	(-0.219)
q70	0.009	(3.266)	0.006	(1.252)
q80	0.012	(3.203)	0.016	(3.331)
q90	0.017	(3.862)	0.010	(1.869)
q95	0.019	(4.930)	0.014	(1.993)

*Notes*: The slopes (t-statistic) of the following regression

$\begin{array}{c} q_{\tau t}\left(A\right)-q_{\tau t}\left(B\right)=\alpha_{\tau }+\beta_{\tau }t+u_{\tau t} \end{array}$

In the first column *A*=Spain, *B*=Globe and in the second *A*=Madrid, *B*=Barcelona.

Summarizing, in this section we describe, measure and test the existence of warming heterogeneity in different regions of the planet. It is important to note that the standard analysis of average temperatures miss this crucial heterogeneity.

## 6 Conclusion

The existence of Global Warming is very well documented in all the scientific reports published by the IPCC. In the last one, the AR6 report (2022), special attention is dedicated to climate change heterogeneity (regional climate). This heterogeneity refers to the non-uniformity of climate change in terms of spatial and temporal dimensions. Our paper proposes a new quantitative methodology to characterize, measure, and test the existence of this heterogeneity, particularly focusing on the evolution of the trend of the whole temperature distribution, rather than just the average temperature. This approach is designed to deal with possible non-stationarities and also with different types of misspecifications (heteroskedasticity, serial correlation, etc). It encompasses both temporal and spatial dimensions in temperature analysis, due to the close alignment between unconditional quantiles and latitude measures.

The findings of our research provide complementary insights to those obtained from climate model simulations. We have observed that the local warming experienced by Spain, known for its climatic diversity, differs significantly from that of the entire globe. In Spain, the upper-temperature quantiles tend to increase more than the lower ones, while the opposite occurs globally. In both cases, the warming process is accelerating over time. Both Spain and the globe experience an amplification effect of an asymmetric nature: there is warming amplification in the lower quantiles of the global temperature, beyond the standard results of the Arctic zone, and in the upper quantiles of Spain. Overall, although there is no warming dominance at the median (mean) level, warming in Spain dominates that of the globe in all the quantiles above the median, while it is dominated at the lower tail of the distribution corresponding to the Arctic region. This places Spain in a challenging warming situation compared to the globe, necessitating stronger regional mitigation-adaptation policies. Therefore, future climate agreements should consider the entire temperature distribution, not just the average, to address this warming heterogeneity effectively.

Any time a novel methodology is proposed, new research issues emerge for future investigation. Among those which have been left out of this paper (some are part of our current research agenda), four points stand out as important:

- There is a clear need for a new non-uniform causal-effect climate change analysis that goes beyond the standard causality in mean. The climate sensitivity parameters may vary across different parts of the temperature distribution, with *CO*2 potentially affecting the lower quantiles more than the upper ones (see [52]). Additionally, the effects of temperature on productivity, income inequality, health, and climate extreme disasters could depend on various distributional characteristics beyond the average. This new causal-effect analysis should be complemented with the construction of a “warming equivalence” metric (in the line of [53]), considering the entire temperature distribution instead of only the average.
- There is a recognized necessity for regional climate forecasts to complement global climate forecasts, particularly due to the existence of climate change heterogeneity. While global forecasts offer valuable information, regional climate forecasts are essential for developing adaptation and planning strategies at the local level.
- In order to enhance efficiency, policies addressing both mitigation and adaptation should consider the existing cross-regional climate heterogeneity (CCH). This heterogeneity implies that such policies should incorporate a common global component as well as a region-specific element. Note that effective regional agreements are easier to achieve than global ones.
- The relationship between the heterogeneity of warming and public awareness of climate change should also be examined.

## Supporting information

S1 Appendix (PDF)
