# Supplementary material for: Climate change heterogeneity: A new quantitative approach
Source: PLoS One. 2025 Jan 28;20(1):e0317208. doi: 10.1371/journal.pone.0317208 (PMC11774374; doi:10.1371/journal.pone.0317208)
Supplement: S1 Appendix — (PDF) [file pone.0317208.s001.pdf]

Appendix: Climate change of Madrid and Barcelona

Madrid-Retiro

Fig A1. Characteristics of temperature data in Madrid-Retiro (AEMET daily data, 1950-2019)

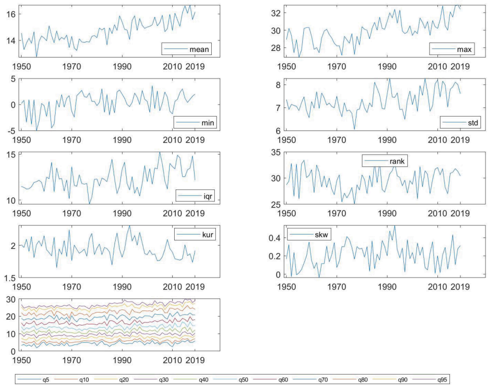

Barcelona-Fabra

Fig A2. Characteristics of temperature data in Barcelona-Fabra (AEMET daily data, 1950-2019)

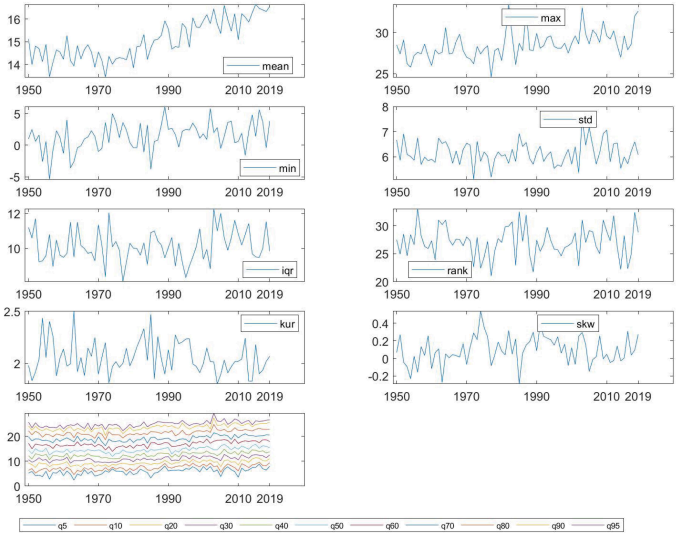

Table A1. Trend acceleration hypothesis (Madrid, daily data, AEMET, 1950-2019)

	**Trend test by periods**		**Acceleration test**
names/periods	1950-2019	1970-2019	1950-2019, 1970-2019
mean	0.0326	0.0447	2.0972
	(0.0000)	(0.0000)	(0.0189)
max	0.0477	0.0636	1.2043
	(0.0000)	(0.0000)	(0.1153)
min	0.0362	0.0087	-1.5077
	(0.0011)	(0.5859)	(0.9330)
std	0.0112	0.0197	2.1160
	(0.0000)	(0.0000)	(0.0181)
iqr	0.0270	0.0399	1.1110
	(0.0000)	(0.0004)	(0.1343)
rank	0.0115	0.0549	2.0160
	(0.3666)	(0.0045)	(0.0229)
kur	-0.0016	-0.0022	-0.4449
	(0.0278)	(0.0660)	(0.6714)
skw	0.0012	-0.0013	-1.7769
	(0.1538)	(0.2695)	(0.9611)
q05	0.0248	0.0183	-0.5712
	(0.0000)	(0.0774)	(0.7156)
q10	0.0220	0.0174	-0.5815
	(0.0000)	(0.0162)	(0.7191)
q20	0.0200	0.0187	-0.1777
	(0.0000)	(0.0099)	(0.5704)
q30	0.0181	0.0235	0.6959
	(0.0000)	(0.0019)	(0.2438)
q40	0.0236	0.0362	1.6625
	(0.0000)	(0.0000)	(0.0494)
q50	0.0299	0.0545	2.8801
	(0.0000)	(0.0000)	(0.0023)
q60	0.0334	0.0604	3.1655
	(0.0000)	(0.0000)	(0.0010)
q70	0.0388	0.0550	1.7385
	(0.0000)	(0.0000)	(0.0422)
q80	0.0519	0.0712	1.9750
	(0.0000)	(0.0000)	(0.0251)
q90	0.0494	0.0687	1.7956
	(0.0000)	(0.0000)	(0.0374)
q95	0.0527	0.0710	1.7839
	(0.0000)	(0.0000)	(0.0383)

Notes: OLS estimates and HAC p-values in parenthesis of the $t_{\beta=0}$ test from regression: $C_t = \alpha + \beta t + u_t$, for two different time periods. For the acceleration hypothesis we run the system: $C_t = \alpha_1 + \beta_1 t + u_t,\ t = 1, ..., s, ..., T, C_t = \alpha_2 + \beta_2 t + u_t,\ t = s+1, ..., T,$ and test the null hypothesis $\beta_2 = \beta_1$ against the alternative $\beta_2 > \beta_1$. We show the value of the t-statistic and its HAC p-value.

Table A2. Co-trending analysis (Madrid-Retiro daily data, AEMET 1950-2019)

Joint hypothesis tests	Wald test	p-value
All quantiles (q05, q10,...,q90, q95)	77.046	0.000
Lower quantiles (q05, q10, q20, q30)	1.360	0.715
Medium quantiles (q40, q50, q60)	2.036	0.361
Upper quantiles (q70, q80, q90, q95)	3.944	0.268
Lower-Medium quantiles (q05, q10, q20, q30, q40, q50, q60)	6.707	0.349
Medium-Upper quantiles (q40, q50, q60, q70, q80, q90, q95)	31.822	0.000
Lower-Upper quantiles (q05, q10, q20,q30, q70, q80, q90, q95)	74.967	0.000
**Spacing hypothesis**	**Trend-coeff.**	**p-value**
q50-q05	0.005	0.505
q95-q50	0.023	0.000
q05-q95	-0.028	0.000
q75-q25 (iqr)	0.027	0.000

Notes: Annual distributional characteristics (quantiles) of temperature. The top panel shows the Wald test of the null hypothesis of equality of trend coefficients for a given set of characteristics. In the bottom panel, the TT is applied to the difference between two representative quantiles.

Table A3. Co-trending analysis (Madrid-Retiro daily data, AEMET, 1970-2019)

Joint hypothesis tests	Wald test	p-value
All quantiles (q05, q10,...,q90, q95)	81.371	0.000
Lower quantiles (q05, q10, q20, q30)	0.424	0.935
Medium quantiles (q40, q50, q60)	8.111	0.017
Upper quantiles (q70, q80, q90, q95)	3.214	0.360
Lower-Medium quantiles (q05, q10, q20, q30, q40, q50, q60)	45.687	0.000
Medium-Upper quantiles (q40, q50, q60, q70, q80, q90, q95)	18.851	0.004
Lower-Upper quantiles (q05, q10, q20,q30, q70, q80, q90, q95)	71.094	0.000
**Spacing hypothesis**	**Trend-coeff.**	**p-value**
q50-q05	0.036	0.004
q95-q50	0.017	0.051
q05-q95	-0.053	0.000
q75-q25 (iqr)	0.040	0.000

Notes: Annual distributional characteristics (quantiles) of temperature. The top panel shows the Wald test of the null hypothesis of equality of trend coefficients for a given set of characteristics. In the bottom panel, the TT is applied to the difference between two representative quantiles.

Table A4. Amplification hypothesis (Madrid daily data, AEMET 1950-2019)

periods/variables	1950-2019	1970-2019	1950-2019	1970-2019
	Inner		Outer	
q05	0.66	0.43	0.83	0.56
	(0.993)	(1.000)	(0.802)	(0.990)
q10	0.58	0.42	0.73	0.54
	(1.000)	(1.000)	(0.974)	(1.000)
q20	0.66	0.53	0.81	0.65
	(1.000)	(1.000)	(0.961)	(0.999)
q30	0.72	0.74	0.94	0.90
	(1.000)	(0.996)	(0.758)	(0.836)
q40	0.90	1.02	1.15	1.21
	(0.887)	(0.436)	(0.072)	(0.041)
q50	1.08	1.29	1.38	1.53
	(0.188)	(0.001)	(0.001)	(0.000)
q60	1.14	1.31	1.44	1.54
	(0.040)	(0.000)	(0.000)	(0.000)
q70	1.22	1.23	1.46	1.38
	(0.012)	(0.019)	(0.000)	(0.002)
q80	1.45	1.36	1.70	1.52
	(0.000)	(0.003)	(0.000)	(0.002)
q90	1.31	1.29	1.48	1.38
	(0.004)	(0.041)	(0.005)	(0.064)
q95	1.31	1.33	1.46	1.39
	(0.001)	(0.021)	(0.007)	(0.073)

Notes: OLS estimates and HAC p-values of the t-statistic of testing $H_0 : \beta_i = 1$ versus $H_1 : \beta_i > 1$ in the regression: $C_{it} = \alpha_i + \beta_i\, mean_t + \epsilon_{it}$. *mean* refers to the average of the Madrid or Spanish temperature distribution for the “inner” and “outer”cases, respectively.

Table A5. Trend acceleration hypothesis (Barcelona, daily data, AEMET, 1950-2019)

	**Trend test by periods**		**Acceleration test**
names/periods	1950-2019	1970-2019	1950-2019, 1970-2019
mean	0.0340	0.0512	3.2979
	(0.0000)	(0.0000)	(0.0006)
max	0.0394	0.0531	0.7280
	(0.0000)	(0.0038)	(0.2339)
min	0.0397	0.0231	-0.7411
	(0.0011)	(0.2654)	(0.7700)
std	0.0013	0.0057	0.9146
	(0.6185)	(0.1787)	(0.1810)
iqr	0.0042	0.0113	0.7351
	(0.4418)	(0.1892)	(0.2318)
rank	-0.0004	0.0300	0.9299
	(0.9806)	(0.3322)	(0.1770)
kur	-0.0013	-0.0018	-0.2693
	(0.1555)	(0.2075)	(0.6060)
skw	0.0011	-0.0022	-1.7869
	(0.2678)	(0.1942)	(0.9619)
q05	0.0374	0.0358	-0.1381
	(0.0000)	(0.0015)	(0.5548)
q10	0.0350	0.0385	0.4361
	(0.0000)	(0.0000)	(0.3317)
q20	0.0317	0.0439	1.7009
	(0.0000)	(0.0000)	(0.0456)
q30	0.0308	0.0488	2.4813
	(0.0000)	(0.0000)	(0.0072)
q40	0.0324	0.0537	2.9244
	(0.0000)	(0.0000)	(0.0020)
q50	0.0325	0.0548	2.7535
	(0.0000)	(0.0000)	(0.0034)
q60	0.0344	0.0636	3.0915
	(0.0000)	(0.0000)	(0.0012)
q70	0.0330	0.0583	2.9241
	(0.0000)	(0.0000)	(0.0020)
q80	0.0357	0.0551	2.4081
	(0.0000)	(0.0000)	(0.0087)
q90	0.0394	0.0567	2.0957
	(0.0000)	(0.0000)	(0.0190)
q95	0.0390	0.0525	1.3435
	(0.0000)	(0.0000)	(0.0907)

Notes: OLS estimates and HAC p-values in parenthesis of the $t_{\beta=0}$ test from regression: $C_t = \alpha + \beta t + u_t$, for two different time periods. For the acceleration hypothesis we run the system: $C_t = \alpha_1 + \beta_1 t + u_t,\ t = 1, ..., s, ..., T, C_t = \alpha_2 + \beta_2 t + u_t,\ t = s+1, ..., T,$ and test the null hypothesis $\beta_2 = \beta_1$ against the alternative $\beta_2 > \beta_1$. We show the value of the t-statistic and its HAC p-value.

Table A6. Co-trending analysis (Barcelona-Fabra daily data, AEMET, 1950-2019)

Joint hypothesis tests	Wald test	p-value
All quantiles (q05, q10,...,q90, q95)	3.368	0.971
Lower quantiles (q05, q10, q20, q30)	1.036	0.792
Medium quantiles (q40, q50, q60)	0.073	0.964
Upper quantiles (q70, q80, q90, q95)	0.784	0.853
Lower-Medium quantiles (q05, q10, q20, q30, q40, q50, q60)	1.171	0.978
Medium-Upper quantiles (q40, q50, q60, q70, q80, q90, q95)	1.901	0.929
Lower-Upper quantiles (q05, q10, q20,q30, q70, q80, q90, q95)	2.969	0.888
Spacing hypothesis	Trend-coeff.	p-value
q50-q05	-0.005	0.528
q95-q50	0.006	0.233
q05-q95	-0.002	0.856
q75-q25 (iqr)	0.004	0.442

Notes: Annual distributional characteristics (quantiles) of temperature. The top panel shows the Wald test of the null hypothesis of equality of trend coefficients for a given set of characteristics. In the bottom panel, the TT is applied to the difference between two representative quantiles.

Table A7. Co-trending analysis (Barcelona-Fabra daily data, AEMET, 1970-2019)

Joint hypothesis tests	Wald test	p-value
All quantiles (q05, q10,...,q90, q95)	13.165	0.215
Lower quantiles (q05, q10, q20, q30)	1.904	0.593
Medium quantiles (q40, q50, q60)	1.267	0.531
Upper quantiles (q70, q80, q90, q95)	0.384	0.943
Lower-Medium quantiles (q05, q10, q20, q30, q40, q50, q60)	10.103	0.120
Medium-Upper quantiles (q40, q50, q60, q70, q80, q90, q95)	1.642	0.949
Lower-Upper quantiles (q05, q10, q20,q30, q70, q80, q90, q95)	9.693	0.207
Spacing hypothesis	Trend-coeff.	p-value
q50-q05	0.019	0.192
q95-q50	-0.002	0.821
q05-q95	-0.017	0.241
q75-q25 (iqr)	0.011	0.189

Notes: Annual distributional characteristics (quantiles) of temperature. The top panel shows the Wald test of the null hypothesis of equality of trend coefficients for a given set of characteristics. In the bottom panel, the TT is applied to the difference between two representative quantiles.

Table A8. Amplification hypothesis (Barcelona daily data, AEMET 1950-2019)

periods/variables	1950-2019	1970-2019	1950-2019	1970-2019
	Inner		Outer	
q05	0.99	0.76	1.19	0.87
	(0.523)	(0.918)	(0.225)	(0.720)
q10	0.90	0.79	1.10	0.94
	(0.824)	(0.980)	(0.263)	(0.668)
q20	0.89	0.85	1.09	1.04
	(0.931)	(0.964)	(0.192)	(0.318)
q30	0.96	0.98	1.22	1.25
	(0.813)	(0.585)	(0.000)	(0.000)
q40	0.99	1.04	1.27	1.33
	(0.570)	(0.300)	(0.000)	(0.000)
q50	1.01	1.07	1.27	1.32
	(0.466)	(0.224)	(0.002)	(0.003)
q60	1.09	1.23	1.29	1.42
	(0.175)	(0.005)	(0.014)	(0.001)
q70	1.09	1.17	1.26	1.31
	(0.128)	(0.012)	(0.022)	(0.008)
q80	1.06	1.04	1.22	1.17
	(0.191)	(0.338)	(0.052)	(0.117)
q90	1.09	1.08	1.22	1.20
	(0.125)	(0.241)	(0.047)	(0.121)
q95	1.06	1.03	1.16	1.12
	(0.304)	(0.432)	(0.192)	(0.298)

Notes: OLS estimates and HAC p-values of the t-statistic of testing $H_0 : \beta_i = 1$ versus $H_1 : \beta_i > 1$ in the regression: $C_{it} = \alpha_i + \beta_i \, mean_t + \epsilon_{it}$. *mean* refers to the average of the Barcelona or Spanish temperature distribution for the "inner" and "outer" cases, respectively.
